# Co-movement between Covid-19 and G20 stock market returns: A time and frequency analysis

**DOI:** 10.1016/j.heliyon.2023.e14195

**Published:** 2023-03-07

**Authors:** Andrew Phiri, Izunna Anyikwa, Clement Moyo

**Affiliations:** Department of Economics, Faculty of Business and Economic Studies, Nelson Mandela University, Port Elizabeth, 6031, South Africa

**Keywords:** COVID-19, Stock markets, G20, DCC-GARCH, Wavelet coherence

## Abstract

In our study, we employ DCC-GARCH and Wavelet coherence analysis to examine the co-movement between global covid-19 indicators (cases, recoveries and deaths) and stock returns of main equity markets in G20 countries using daily data spanning between February 2, 2020 and August 28, 2021. Our empirical results show that the co-movement between COVID-19 and G20 stock returns has been switching between negative and positive correlations across the entire time window. The wavelet coherence analysis further reveal that negative (positive) co-movements predominantly exist as lower (higher frequencies) for cases and deaths and are more mixed for recoveries. The findings also show that the short-frequency components correspond to periods around the initial announcement of the initial pandemic and also around the announced of subsequent variants of the COVID-19 virus. Policy and market implications from our study are also discussed.

## 1 Introduction

What began as an outbreak of the COVID-19 virus in Wuhan, China in December 2019, quickly escalated into a global pandemic with the virus growing at an exponential rate and spreading to all regions across the globe. On March 11th^,^ 2020, the coronavirus outbreak was declared as official global health crisis by the World Health Organization (WHO) and the severity of the pandemic forced governments worldwide to declare ‘state of emergency’ and to focus all efforts in trying to ‘flatten the curve’ of viral infections by the ‘shutting-down’ of major cities. Due to the initial absence of a clinically approved vaccine, non-pharmaceutical interventions were undertaken by government worldwide including travel bans, social isolation and distancing as well as closure of schools and non-essential business, all which took a major toll on economic activity via supply-chains, the financial stability of companies, government fiscal balances, trade activity and household consumption [1]. The ‘shocks’ from these abrupt shutdowns have been likened to those experienced during periods of wars and natural disasters [2,3]. Barro et al. [4] finds that whilst current advances in public health care and non-pharmaceutical intervention have lowered the chances of the COVID-19 mortalities reaching those reported for previous pandemics, this has come at more severe economic repercussions and has required government interventions primarily in the form stimulus packages, debt relief packages, and other market interventions [5,6]. Moreover, the uncertainty surrounding the pandemic, led to much distortion in stock markets, as market participants were driven by ‘fear’ [7,8] and sought to diversify their investments and portfolios into safer assets, commodities [9,10] and even cryptocurrencies [11].

Notably, stock exchanges in G20 countries were amongst the first economic institutions to absorb the adverse shocks of the COVID-19 pandemic, which is not surprising considering the size and interconnectedness of these markets [12] as well as the fact that these countries are the epicentres of infections and mortality rates. For instance, the stock prices in Chinese markets fell by as much as 40% days after the first recorded death in Wuhan on December 1st [13]. In March 2020, the circuit breaker mechanism in US stock markets was activated four times in 10 days which halted all trading activity and during this period stock prices plummeted by as much as 35% [14]. Similar trading halt occurred in major stock exchanges in Mexico, Canada, Brazil and South Korea after the key indices in these markets fell below the −7% ‘trigger benchmark’ [12]. Moreover, the main stock market indices in other industrialized members of G20 countries such as the EURO STOXX, the UK's FTSE, France's CAC40, the Spanish IBEX35, the Australian S&P/ASX 200, Japan's NIKKEI225, Germany's DAX, dropped more than 30% [[15], [16], [17]] whilst the Japanese stock markets took a further plunge following the cancellation of the Olympic winter games [18]. Emerging economies amongst the G20 nations such as BRICS (Brazil, Russia, India, China, South Africa) and MINT (Mexico, Indonesia and Turkey) countries were also severely affected by heavy portfolio outflows of bonds and equities into safe-haven assets, leading to distortions in currency and equity markets [19]. Other emerging G20 oil-producing economies such as Saudi Arabia and Russia were engaged in a geopolitically induced oil price war which had not only collapsed the US WTI prices but also had spillover effects in currency and stock markets in these economies [20].

**Fig. 1:**
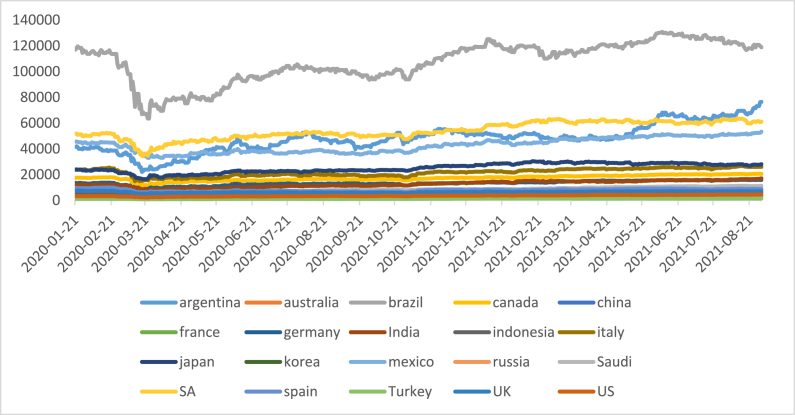
Stock indices in G20 equity market

**Fig. 2:**
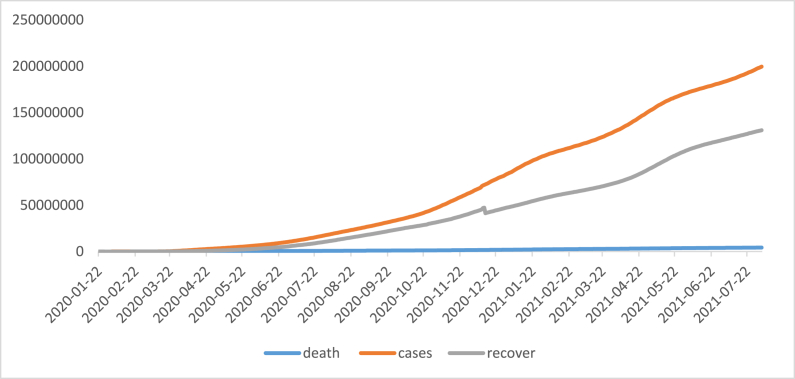
COVID-19 cases, recoveries and deaths

Remarkably, stock markets in G20 economies have recovered exceptionally well since April 2020 (see Fig. 1), with most markets reaching new highs at the end of January 2021. Whilst some recent studies have attributed the recovery in stock markets to government intervention in the earlier stages of the pandemic [5,6], what remains surprising is that pandemic has gotten worse in terms of cases and deaths (see Fig. 2), and yet stock markets have continued to perform well with minimal subsequent government intervention. According to the Black Swan theory, disaster events cause unprecedented shock and fear in markets resulting in panic trading of shares and irrational behaviour by market participants. This is contrary to the efficient market hypothesis of Fama [21] which argues that market participants are rational agents, and no specific group of investors can use historic, public or private information to beat the market and make abnormal returns. Notably, opponents of the EHM, with Robert Shiller at the forefront, have argued that the COVID-19 pandemic sets an example of a situation whereby market participants have exhibited irrational behaviour which is in violation of the market efficiency hypothesis. Moreover, the adaptive market hypothesis of Lo [22] implies that market efficiency is subject to evolutionary factors in financial markets and, interestingly, some recent studies have provided evidence in favour of the adaptive market hypothesis during the pandemic, albeit demonstrating this by providing evidence of time-varying weak-form market (in)efficiency during the COVID-19 period [14,23].

In our study, we investigate the extent to which information derived from COVID-19 health statistics correlates with stock returns in G20 equity markets which is a hypothesis in support of the Black Swan theory and the adaptive market hypothesis and yet in violation of the semi-weak form EMH. Whilst we are aware of the emerging literature which has recently examined the co-movement between COVID-19 health statistics and equity returns in either individual G20 countries ([24,25] for the US [26]; for Canada [27,28]; for China [29]; for Saudi Arabia [30,31]; for Australia), a group of G20 countries ([23] for all G20 countries excluding South Africa [15]; for 7 of the G20 countries namely China, Italy, South Korea, Spain, Germany, Japan and the US) or the entire group [12], our study makes two unique contributions to the growing literature.

Firstly, in differing from most previous studies we make use the dynamic conditional correlation generalized autoregressive conditional heteroskedasticity (DCC-GARCH) model and continuous wavelet transforms to examine the dynamic correlations between COVID-19 health statistics and G20 stock returns. Whilst the DCC-GARCH technique models the time-varying co-movement in the volatility between the variables, the continuous wavelet coherence tools model the co-movements between the series within a time-frequency space. Notably, the wavelet methodology differs from previous methodologies used in the literature such as the vector autoregressive (VAR) model [26], dynamic regression analysis [32,33], spatial econometric techniques [12], detrended cross correlation analysis (DCCA) [23], which are strictly time-domain techniques and ignore frequency-varying correlations amongst the variables. Distinguishing between these different frequency components is important for capturing for the heterogenous activity of different market participants who base their decisions across different frequency horizons. In practice, speculative traders and myopic investors would be interested in obtaining public information related to shorter time horizons where the time series data is characterized by higher frequency oscillations whereas long-term or safer investors would be more concerned with long-term or lower frequency variations between COVID-19 information and stock market returns.

Secondly, our study makes use of a longer time series than those used in previous studies. To the best of our knowledge, the previous studies employ time series which extends at furthest until September 2020 (see Ref. [34]) and does not cover the 2021 period, hence excluding notable global events such as the US Presidential elections, the capitol insurrection attacks, the rollout of vaccines as well as the more recent Kabul airport bombings following the Taliban's hostile takeover of the Afghanistan government, all which have had significant impact on global equity markets. Moreover, using a longer series enables us to get a clearer picture of the co-movement between COVID-19 and equity returns across the periods covering the announcement of newer variants of the disease (Alpha, Beta, Gamma and Delta) between December 18, 2020 and April 4, 2021.

All-in-all, the findings from our study can be summarized in three points. Firstly, the DCC-GARCH model shows that volatility co-movement between the series differs amongst different measures of COVID-19. For instance, volatility co-movement using COVID-19 cases was extremely high until mid-March and smoothened afterwards whereas volatility between COVID-19 recoveries and deaths has shown strong co-movement across or entire sample period frequently switching between negative and positive correlations between the series. Secondly, the wavelet coherence analysis reveals evidence of ‘sign switching’ between negative and positive correlations in the co-movement between COVID-19 statistics and G20 stock market returns at different frequency level, with positive correlations dominating lower frequency and negative correlations dominating higher frequencies. Lastly, the dynamic co-movements between the COVID-19 statistics and equity returns in the different G20 countries do not differ much from each other which fits well with narrative of the interconnectedness of the equity markets with strong contagion effects during slumps and recovery [12].

Notably, our findings add new knowledge to the current body of literature which appears to be in consensus of negative (positive) impact of COVID-19 cases and deaths (recoveries) on G20 stock returns [23,25,26,32,[35], [36], [37], [38]]. Our study demonstrates that the strict reliance on time domain econometric techniques masks important frequency correlations which exist amongst the data which, in turn, is important to understanding the varying market efficiency amongst the stock exchanges during the COVID-19 pandemic. Also, in contradiction to the ongoing literature, we find that COVID-19 has had predictive power over stock returns over the entire COVID sample period particularly for recovery and death statistics. Our results counter the argument that predictive power of COVID-19 statistics on stock returns were short-lived during the pandemic [15,23]. From a theoretical perspective, our study fails to find support in strict adherence to the semi-strong EMH and is rather in conformity to AMH as well as the Black Swan theory. From a market perspective, market efficiency is found to be most comprised during the initial announcement of the pandemic in March 2020 as well as during the announcement of the Alpha and Beta variant in December 2020 and the more recent Delta variant in April 2021, particularly for COVID-19 recovery statistics. Finally, from a policy perspective, governing health bodies may be interested to note that whilst the stock market may be distinct from the economy (as Paul Krugman crudely puts it), our study suggests that markets are not distinct from the global health status, at least not during periods of the pandemic, and therefore health policies and outcomes are pivotal in enhancing the informational efficiency of stock markets in these peculiar times.

We structure the rest of the paper as follows. Section 2 provides a review of the associated literature. Section 3 outlines the methodology used in our empirical analysis. Section 4 presents the data. Section 5 presents empirical findings. Section 6 concludes the study.

## 2 Literature review

The COVID-19 pandemic is the deadliest wave of viral infection our current generation has faced and there is increasing scientific consensus that the pandemic satisfies all three attributes of a Black Swan event identified by Taleb [39] i.e. “… *First, it is an outlier, as it lies outside the realm of regular expectation. Second, it carries an extreme impact. Lastly, in spite of its outlier status, human nature makes us concoct explanations for its occurrence* …” [17,40,41]. Whilst black swan episodes are manifested through various unanticipated, rare and severe events such as wars, economic depressions, hyperinflation, financial crisis, terrorist incidents, chemical disasters, natural disasters and environmental catastrophes [42,43], the COVID-19 pandemic has been categorized as a disease-induced ‘black swan event’ along with other previous viral outbreaks such as Bird Flu, SARS, Ebola and EMRS [41].

Disease-induced ‘black swan events’ tend to produce one-time shocks on financial and equity markets which causes investors to diversify their portfolio holdings as a means of mitigating the risks associated with heightened uncertainty whilst risk-takers, such as speculators, tend to seek for short-term gains amidst financial turmoil [41,44]. The accumulation of these ‘fear and greed’ factors results in ‘irrational behaviour’ of market participants during these ‘black swan events’ and this contradicts the self-correcting market behaviour induced through rational expectations as advocated by the efficient market hypothesis (EMH) of Fama [21]. A more encompassing theory explaining ‘black-swan events’ as an evolutionary phenomenon is the adaptive market hypothesis of Lo [22] which replaces the assumption of rational expectations with unbounded rationality or hyperrational expectations and assumes that market participants develop adaptive traits to enhance their ‘survival’ in financial markets. The enhanced ‘survival behaviour’ of investors during black swan events tends to shift equity prices away from their fundamental values, which then offers market participants arbitrage opportunities to gain abnormal profits by predicting future returns of stock exchanges using historic or publicly available information [14].

There have been many empirical studies which have examined the impact of disease-induced black swan events on stock market performance and for convenience sake, we segregate the discussion of the related literature into two parts. Firstly, we review studies which have investigated the impact of previous viral epidemics, such as Bird Flu, SARS and Ebola, on stock markets performance and we note that most of these studies focus on Asian economies. Secondly, we review more recent studies which have examined the impact of the COVID-19 pandemic on stock exchanges and most of this empirical literature is centred around G20 countries.

Under the first strand of empirical literature, most existing studies focused on the impact of the SARS and Bird Flu outbreaks on Asian equity markets whilst the remaining studies focused on the effect of the Ebola virus on African stock exchanges. For instance, Nippani and Washer [45] find a negative impact of the SARS stock exchanges in Chinese and Vietnamese stock markets whereas no effect is found stock markets in Canada, Hong Kong, Indonesia, Philippines, Singapore and Thailand. Conversely, Chen et al. [46] find evidence that SARS negatively affected equity markets in China, Hong Kong, Singapore, Taiwan and Japan, and weakened integration amongst these markets. Other empirical studies showed that SARS and Bird Flu epidemics had negative effects on Chinese housing markets [47], hotel sector [48], tourism, wholesale and retail sectors [47] and yet positive effects were found for biotechnology and pharmaceutical companies in China and Vietnam [47,[49], [50], [51]]. For the Ebola virus, Del Guidice and Paltrinieri [52] found that the outbreak caused investors to withdraw their savings in mutual funds which caused a decline in equity capital injections into African stock markets. Moreover, Ichev and Marinč [53] found that Ebola virus had severe negative effects on stock returns for US and European companies with exposure to West African countries (the birthplace of the disease).

Under second strand of literature, the associated studies examined the impact of COVID-19 health statistics on equity returns and notably this literature is still developing as the pandemic is currently an ongoing phenomenon. Whilst initial empirical studies found an absolute negative effect of COVID-19 cases and deaths on Chinese and US stock exchanges using data extending to the end of March 2020 [26,32,35] for Canada and the US), as more data become available subsequent studies have found that stock markets have reacted less to the growing number of COVID-19 statistics [12,15,36,38]. Some other studies identify discrepancies on the impact of the pandemic on stock exchanges in different regions of the world. For instance, Topcu and Gulal [33] finds that the impact of the pandemic is more severe in Asian markets, whereas European markets were least affected. Okorie and Lin [23] find that adverse effects of virus in stock exchanges for 32 industrialized economies is more significant in economies with higher cases of COVID-19 in comparison to countries with lower cases. Moreover, Salman and Ali [18] find that the adverse effect of the pandemic on GCC countries relatively smaller compared to that of global stock markets; Ashraf [38] find a negative response of stock returns is greater for higher levels of uncertainty aversion of investors whereas Harjoto et al. [37] finds that find that emerging countries with smaller market size where most adversely affected by the pandemic. Lastly, there exists some industry-level evidence which has shown that the pandemic has had positive impacts for information technology and medicine manufacturing sectors in the Chinese markets [27], health, information technology and consumer staples sectors in Australia [30] and natural gas, food, healthcare and software development sectors in the US [25]; as well as for listed companies with the largest market capitalization and profitability in Australian stock exchanges [31].

As detailed in the introduction, our study contributes to the line of recent literature by investigating the co-movements between the stock market and coronavirus pandemic for the G20 countries using extended time series data and more advanced econometric techniques, namely, the DCC-GARCH and Wavelet coherence techniques, which are used to provide new insights into the coronavirus-equity returns relationship in light of more recent health and geopolitical developments. These methods are outlined in the following section of the paper.

## 3 Methodology

The empirical approach employed in this paper composed of two frameworks, the DCC-GARCH approach and Wavelet Analysis approach. These two approaches are discussed below.

### 3.1 DCC-GARCH model

The importance of volatility and correlation in understanding co-movements of stock markets have been acknowledged in the literature. In particular, studies have shown that stock market volatility and correlation tend to increase during periods of greater market uncertainty [54]. To capture such a phenomenon, the DCC-GARCH model proposed Engle [55] is used in this study to analyse the behaviour of volatility and correlation of stock markets in relation to COVID-19. The DCC-GARCH framework for this study is specified as:

(1) $$r_{i,t}=\mu_{i,t}+\varphi_{i}r_{i,t-1}+\varepsilon_{i,t}\;\varepsilon_{t}\Omega_{t-1}∼N\left(0,H_{t}\right)$$

where r is the stock market returns and $\varepsilon_{i,t}$ is the innovation which is conditional on the information set available at time t−1 is denoted by $\varepsilon_{t}\Omega_{t-1}$ and $H_{t}$ is the conditional variance-covariance matrix. The conditional variance-covariance matrix, $H_{t}\text{,}$ can be written as:

(2) $$H_{t}=D_{t}R_{t}D_{t}$$

where $H_{t}$ is also the conditional correlation estimator, $D_{t}$ is an (n×n) diagonal matrix of time varying conditional standard deviations of return in the mean equation (1) at time t from the univariate GARCH (1,1) model. $D_{t}=diag\left(\sqrt{h_{i,t}},\ldots ,\sqrt{h_{i,t}}\right)$, Here, $h_{i,t}$ represents the time-varying conditional volatility of return for a given stock market which is assumed to follow a GARCH (1,1) model as provided below:

(3) $$h_{i,t}=\omega_{i}+\beta_{i}h_{i,t-1}+\alpha_{i}\varepsilon_{i,t-1}^{2}$$

where $\omega_{i}$ is a constant term, $\alpha_{i}$ and $\beta_{i}$ represent the ARCH and GARCH effects respectively. A positive $\alpha_{i}$ indicates existence of volatility clustering while $\beta_{i}$ represent persistence behaviour. The sum of $\alpha_{i}$ and $\beta_{i}$ indicates the persistency of volatility shock.

From equation (2), $R_{t}$ is a time-varying conditional correlation matrix and it is computed using the standardized residuals obtained from GARCH (1,1) estimation as:

(4) $$R_{t}=Q_{t}^{*-1}Q_{t}Q_{t}^{*-1}$$

(5) $$Q_{t}=\left(1-\alpha -b\right)\bar{Q}+\alpha \varepsilon_{t-1}\varepsilon_{t-1}^{T}+bQ_{t-1}$$

where $\bar{Q}=Cov\left[{\varepsilon_{t}\varepsilon_{t}}^{T}\right]$ = $E\left[{\varepsilon_{t}\varepsilon_{t}}^{T}\right]$ is the unconditional covariance matrix of the standardized residuals and $Q_{t}^{*}=diag\;\left(\sqrt{q_{ij,t}}\right)\text{.}$ Engle [55] suggests that the time-vary conditional correlation $\left(q_{ij,t}\right)$ for any two variables can be written as

(6) ρij,t=qij,tqii,tqjj,t

Hence, the conditional correlation between the variables is computed as:

(7) $$\rho_{ij,t}=\frac{\left(1-\alpha -b\right){\bar{q}}_{ij}+\alpha \varepsilon_{i,t-1}\varepsilon_{j,t-1}+bq_{ij,t-1}}{\sqrt{\left((1-\alpha -b\right){\bar{q}}_{ii}+\alpha \varepsilon_{i,t-1}^{2}+bq_{ii,t-1})}\left(\left(1-\alpha -b\right){\bar{q}}_{jj}+\alpha \varepsilon_{j,t-1}^{2}+bq_{jj,t-1}\right)}$$

where $\rho_{ij,t}$ is the conditional correlation between stock return series for markets i and number of COVID-19 infections (j) at time t. Specifically, $\rho_{ij,t}$ measures the direction and strength of correlation between the two variables. If $\rho_{ij,t}$ is positive, the correlation between the two variables is rising and moving is the same direction. But, when $\rho_{ij,t}$ is negative the correlation is declining, and both variables are moving in opposite direction. The parameters in equation (7) are estimated using the quasi-maximum likelihood method (QML).

### 3.2 Wavelet analysis

The second approach used in this paper is the wavelet analysis which enables simultaneous analysis of co-movement between stock markets and COVID-19 infections. This approach offers a way of analysing localised variations of power within time series. As such, it provides a framework to determine the level of interdependencies between two time series variables in both frequency and time spaces [56]. In addition, the model captures the possible dynamic changes in the relationship by accounting for both short-run and long-run movements. Specifically, this paper employed the wavelet coherence (WTC) and cross-wavelet phase angle (phase difference) to analyse the dependencies between stock market and COVID-19 infections. This framework is briefly presented below.

By definition, a continuous wavelet transform (CWT) with respect to the wavelet ψ is a function of two variables $W_{x}\left(\tau ,s\right)$ can be expressed as:

(8) $$\psi_{\tau ,s}\left(t\right)=\frac{1}{\sqrt{s}}\;\psi \left(\frac{t-\tau }{s}\right)$$

Where the wavelet functions ψ(t) depends on time parameter t, 1s is a normalization factor ensuring the unit variance of the wavelet ${\left\|\psi_{\tau ,s}\right\|}^{2}=1$, τ is the location parameter that gives information about the exact position of the wavelet and s is the scale dilatation parameter of the wavelet. It defines how the wavelet is stretched. In order to measure the amplitude of a given time series or variance of the series at each time and scale, the wavelet power spectrum (WPS) is defined as: ${WPS}_{p}\left(\tau ,s\right)={\left|W_{p}\left(\tau ,s\right)\right|}^{2}$. This gives a measure of the variance distribution of the series in the time scale and frequency plane.

Following Aguiar-Conraria and Soares [57], and Dash and Maitra [58], we specify wavelet coherence for two time series x(t) and y(t) with the wavelet transforms $W_{x}\left(\tau ,s\right)$ and $W_{y}\left(\tau ,s\right)$ and the cross-wavelet spectrum $W_{xy}\left(\tau ,s\right)=W_{x}\left(\tau ,s\right)W_{y}^{*}\left(\tau ,s\right)\text{,}$ as

(9) $$R^{2}\left(\tau ,s\right)=\frac{{\left|S\left(s^{-1}W_{xy}\left(\tau ,s\right)\right)\right|}^{2}}{S\left(s^{-1}{\left|W_{x}\left(\tau ,s\right)\right|}^{2}\right)S\left(s^{-1}{\left|W_{y}\left(\tau ,s\right)\right|}^{2}\right)}$$

Where S denotes the smoothing operator both over time and scale, with $0\leq R^{2}\left(\tau ,s\right)\leq 1\text{.}$ A high (low) value of $R^{2}$ indicates higher (lower) level of co-movement between the two variables. The graphical plot of the wavelet coherence will show the time and frequency space representing the co-movement of the two variables across time and scale.

## 4 Data and results

In this paper, we employ daily closing stock price index of G20 countries and six data relating to daily movement in COVID-19 outbreak, namely total number of infections worldwide (cases), number of deaths worldwide (death) and number of recoveries worldwide (recovery). The daily data span the period of four months start from February 2, 2020 to August 31, 2021. The stock price index data were obtained from Investing.com website while the COVID-19 data is sourced from Worldometer database. The daily stock price index is converted to daily stock returns using the continuous compounding technique as: $r_{t}=100\times \ln\left(\frac{P_{t}}{P_{t-1}}\right)$.

Table 1 reports the basic statistics of the daily stock returns and COVID-19 proxies. The reports show that on average, approximately 8.5% cases of coronavirus infections are recorded globally on a daily basis. While the average daily recovery rate stands at 8.4%, the average number of deaths is 10.2% daily over the period. Another notable fact emerging from the table is that the average stock returns are negative for all G20 stock markets. Specifically, markets such as Brazil, France, Italy and Spain recorded the largest losses with the average daily returns among these markets ranging from −0.37% to −0.29. This is expected given that these countries were among the worst affected countries apart from the U.S. The skewness coefficients indicate high tendency of realising negative stock returns than positive ones. The high level of kurtosis, particularly for the stock returns, indicate that extreme change in these stock returns often occur. Moreover, Jarque-Bera test rejected the null hypothesis of a normal distribution for all the variables at 1% level of significance.

Furthermore, we employ the augmented-Dickey-Fuller (ADF) and Phillips and Perron (PP) tests and find that all the series are I(1) apart from Death and Indonesia. The Ljung-Box, LB(10) and LB-sq (10) show the existence of serial correlation in a number of series. Similarly, the we find the existence of ARCH effects in a number of stock markets. Given the existence of ARCH effect is the stock return series, this allows us to estimate the DCC-GARCH model. In addition, the unconditional correlation reported in Table 2 shows the existence of positive relationship between the G20 stock returns and COVID-19 (except for China and Indonesia for cases and Indonesia for recoveries). However, studies have cautioned against the use of unconditional correlation as a measure of co-movement, particularly in the presence of heteroskedasticity [59]. Hence, there is a need to examine the co-movements using more dynamic models such as DCC-GARCH and Wavelet models.

## 5 Empirical results

This empirical result section is divided into two subsections in order to facilitate comparison of the results from the two frameworks used to analyse the co-movement of stock market returns and COVID-19 in G20 countries. While the first part of the section focuses on the analysis of DCC-GARCH (1,1) results, the second part presents and analyzed the results from the wavelet analysis.

### 5.1 DCC-GARCH result

We employed the DCC-GARCH (1,1) approach to consider the dynamic nature of the co-movement between the G20 stock market returns and COVID-19. The main advantage of the DCC-GARCH model is that it allows the condition volatility and correlations to vary over time, which accounts for the dynamic behaviour of stock markets. Table 3 displays the results of DCC-GARCH (1, 1). As shown in the table, the estimated DCC-GARCH parameters are statistically significant, particularly the ARCH (α) and GARCH (β) coefficients, suggesting a good deal of persistence in the conditional volatility. Moreover, the a+b is less than unity (except for China), indicating the stability of the estimated DCC model. But, given that the sum is closed to unity, it implies persistence in the conditional correlation. Additionally, the report in Table 4 shows that apart from Italy there is no further presence of ARCH effect in the series. This finding supports the robustness of the estimated DCC-GARCH (1,1) to the data series.

**Fig. 3:**
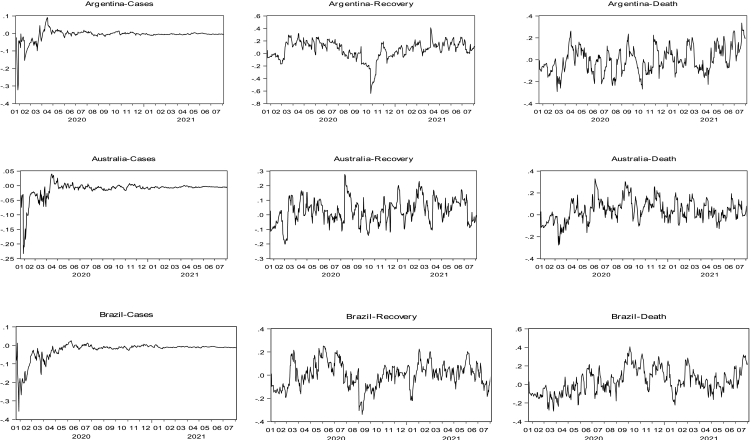

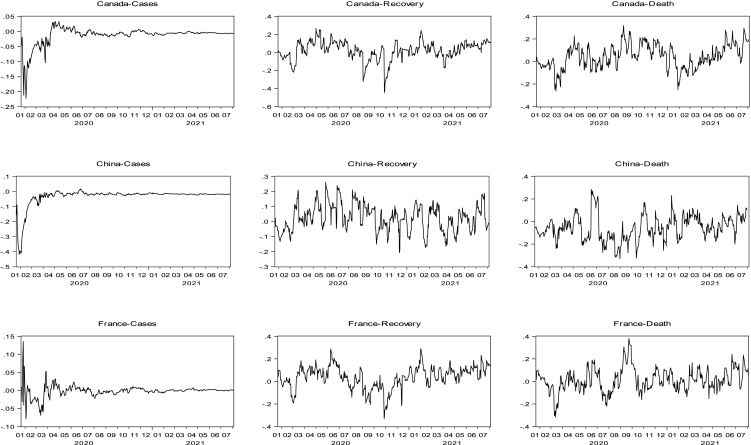

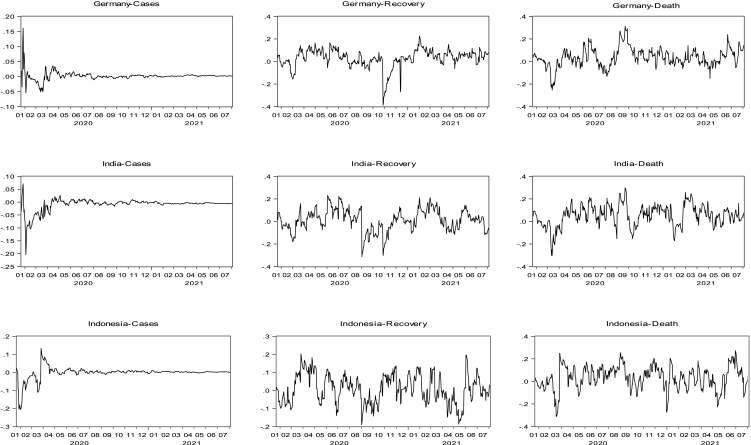

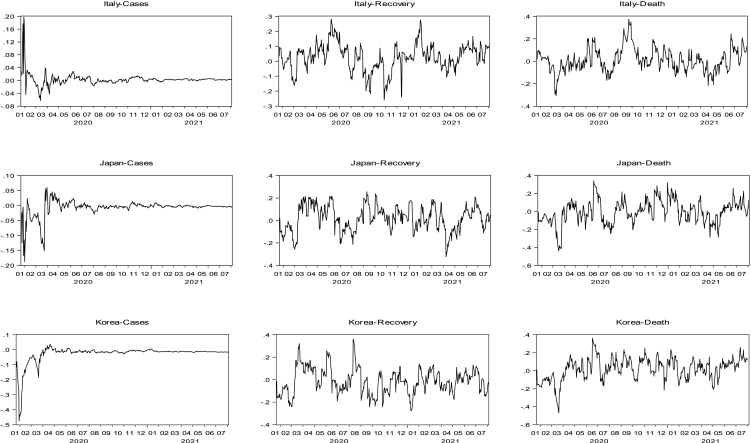

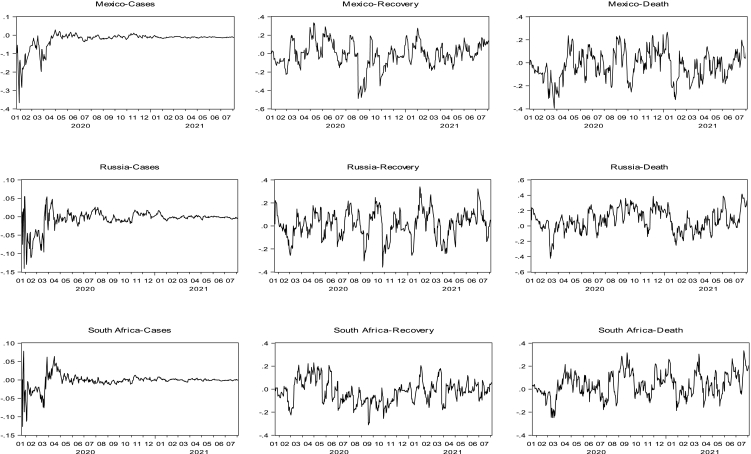

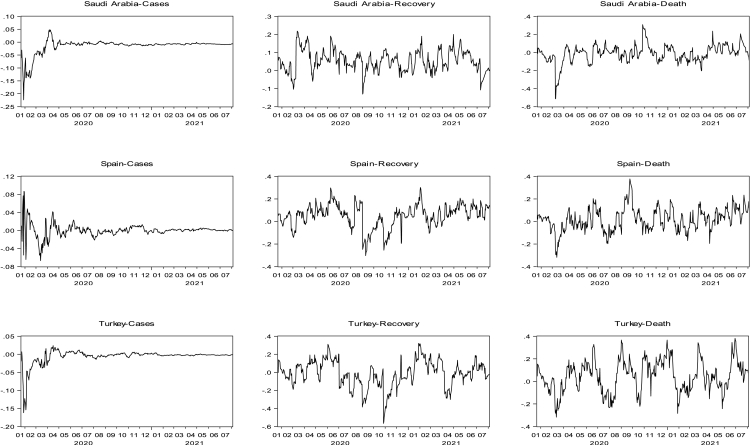

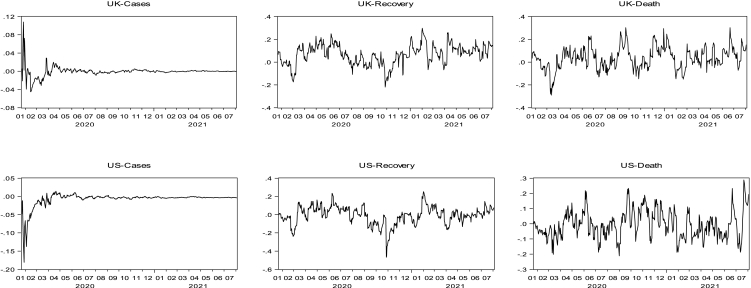
DCC between the G20 stock markets and COVID-19

To get a visual impression of the dynamic behaviour of relationship between the G20 stock returns and COVID-19, we plot the dynamic co-movements of the conditional correlations over the sample period in Fig. 3. The figure shows that the dynamic co-variability between the G20 stock returns and cases of coronavirus infections are switching between negative and positive co-movements and this is clearly visible in the first 4 months of the pandemic. Thereafter, the movement between cases and equity returns predominantly revolves around zero trend. When the number of recoveries and deaths are considered with the G20 stock returns, the switching dynamics are still observed albeit across the entire time window frame. To further provide more information on the behaviour of the dynamic co-movements, Table 4 reports the average value of the dynamic conditional correlation. The report in the table collaborates the findings from Fig. 3. Specifically, the table shows that the G20 stock market returns are negatively correlated with cases of coronavirus infection (except Germany and Italy). This finding implies that a negative movement is observed in the stock returns as more cases of coronavirus infections are discovered. The finding is consistent with literature on the negative effect of pandemic on stock market performance. As expected, recoveries are associated with positive movement in stock returns. Surprisingly, death from the virus is associated with positive movement of stock returns. This finding runs counter to the expectation given that negative news is usual associated decline in stock market returns.

### 5.2 Wavelet analysis

**Fig. 4:**
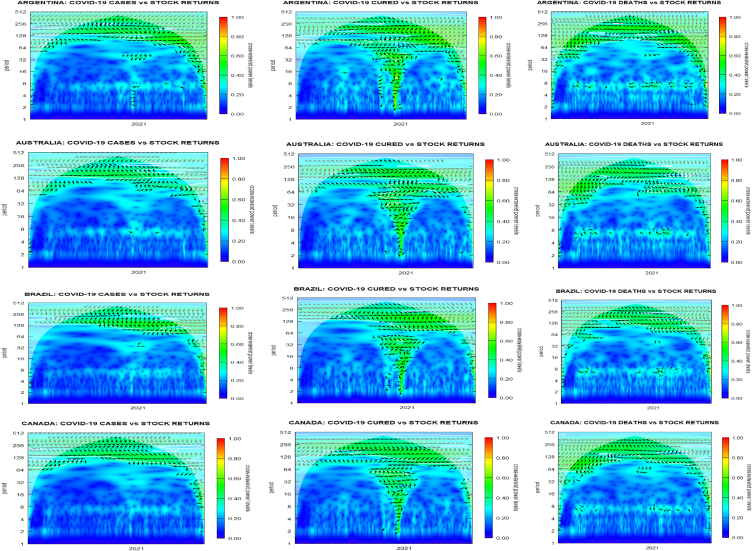

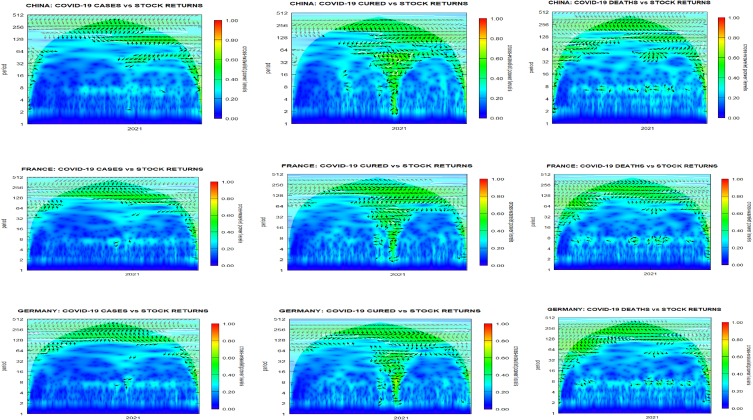

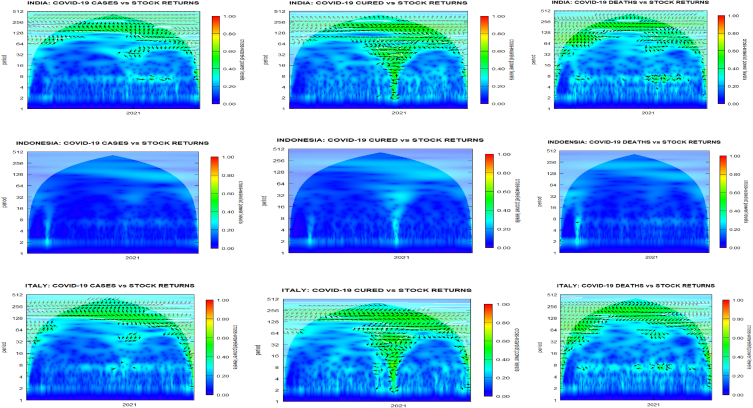

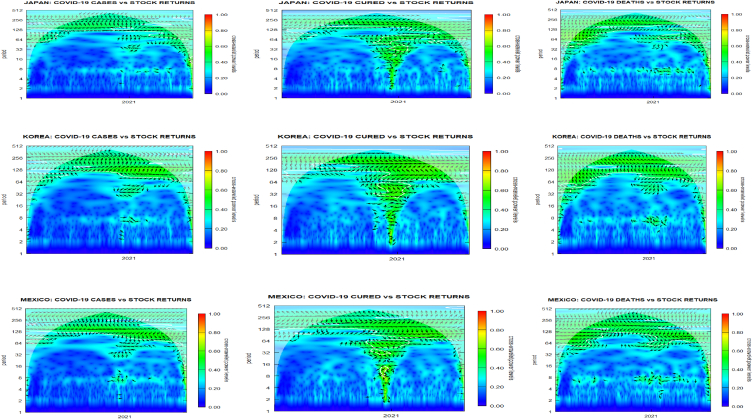

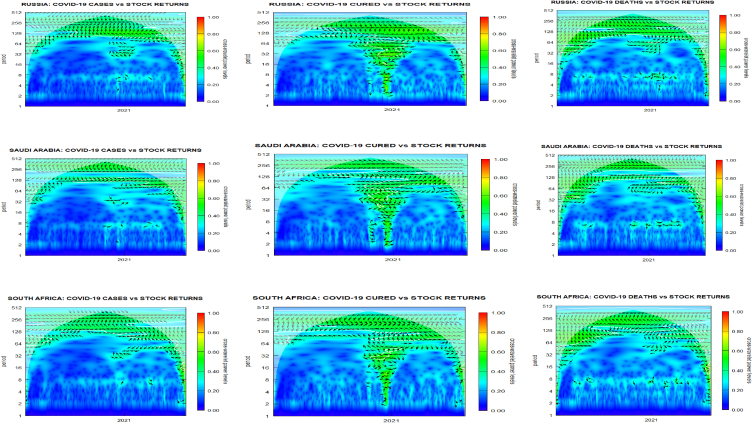

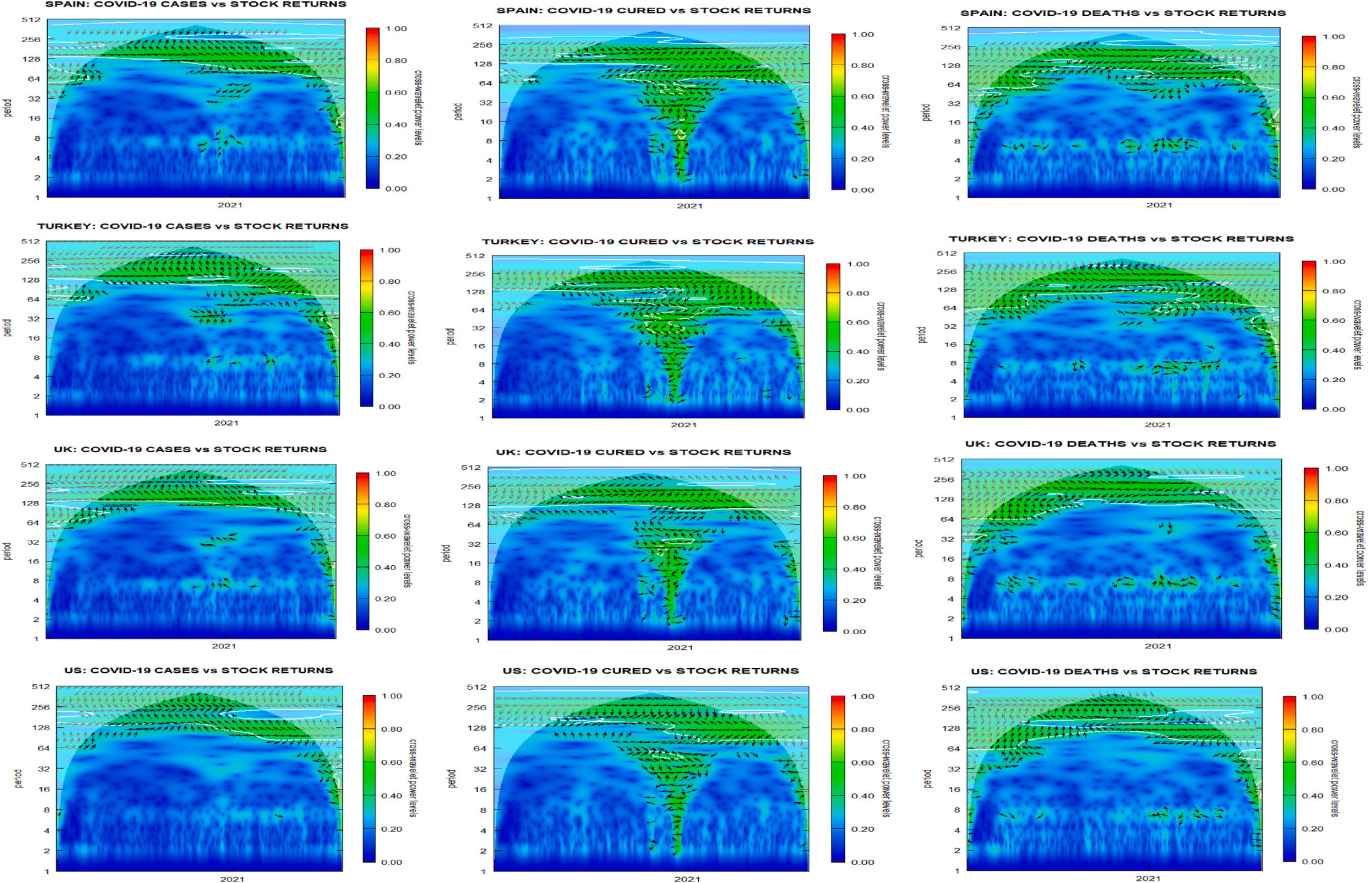
Wavelet coherence plots between COVID-19 statistics and G20 stock market returns

Fig. 4 presents the wavelet coherence plots between covid-19 measures (cases, cures and deaths) and stock returns for the G20 countries. These cross-wavelet transforms plots provide correlation measures between the pair of time series across three dimensions, namely, time, frequency and time-scale cross wavelet coherence levels. Firstly, the time dimension is captured along the horizontal axis of the plots and captures the daily data from February 2, 2020 to September 31, 2021. Secondly, the frequency dimension is measured along the vertical axis of the plots which is converted into daily units, with the frequency ranging from 1-day (highest frequency) to 512-day (lowest frequency) cycles. Thirdly, the time-scale wavelet coherence levels are captured are represented by a colour spectrum, with the blue shading indicating low coherence levels, green/yellow shading indicating medium coherence and red shading indicating high coherence. Within the diagrams, significant coherence is identified by regions surrounded by a faint white contour line which represents a 5% critical level. The arrows themselves, represent the phase difference dynamics between the time series, are represented by arrows and this provide additional information concerning the synchronization between the variables. The right (left) pointed arrow denotes phase-in (phase-out) which can be interpreted as a positive (negative) correlation between the series. The arrows pointing to the north-east or south-west indicate that the coronavirus leads the stock market returns and all arrows facing north-west, or south-east implies that stock returns are leading market returns. The expected direction of the ‘arrows’ is for them to point left, right, north-east or south-west, which all imply lead-lag relationship from coronavirus indicators to stock market returns and not vice versa.

From the onset, it is interesting to note that dynamic correlations between the COVID-19 statistics and equity market returns are similar across all G20 member states across a time-frequency domain which is expected considering the observed interconnectedness of the G20 equity markets during the pandemic [12]. It is also interesting to note that significant led-lag synchronizations from the different COVID-19 statistics to equity returns exist across the entire time window albeit at different frequency oscillations. Most of the significant co-movement is found within regions of between 64 and 512 cycle days which are rather low frequency components. However, we observe episodes of much higher frequency oscillations in three instances. Firstly, for COVID-19 cases most of the higher frequency covariation is observed at around March 2021, in which frequency oscillations increase to as high as 24-day cycles by July 2021. Secondly, for COVID-19 cures the higher frequency covariations are found i) around December 2020 which coincides with the announcement of the Alpha and Beta variants as well as the peak of the second wave ii) from April 2021 onwards which corresponds to period when the delta variant was announced. Lastly, for COVID-deaths most higher frequency covariation is found during the early stages of the pandemic around March 2020 as well as towards the end of the sample around June 2021.

From the phase difference dynamics within the wavelet coherence the plots, we find that the cases and deaths series are predominantly anti-phase (negative) at lower frequency and more in-phase (positive) at high frequency oscillations whereas we observe mixed patterns for recoveries particularly at higher cycles. In linking these findings with the present literature which predominantly finds a negative co-relationship between COVID cases/death statistics and equity returns in G20 markets [25,26,32,33,[35], [36], [37], [38]], our results reveal that these negative oscillations mainly occur at lower, smoother frequencies. Moreover, our findings refute the claims that the pandemic has become less correlated with equity markets over time [15,23]. Our findings also show that whilst higher frequency oscillations appear and disappear across the entire time window, the low-frequency covariations have consistently existed throughout the entire pandemic. All-in-all, these findings are more-or-less in line with those previously obtained from the DCC-GARCH analysis in which the covariation between COVID-19 statistics and equity returns in G20 markets has been switching between negative and positive across the entire time window.

Altogether, our findings provide insights to how stock market returns, and volatility respond to COVID-19 statistics and hence imply that the investors and portfolio managers can use information publicly available COVID-19 statistics to predict stock market returns. However, our findings show that show that the COVID statistics have different impact on stock market activity at different frequency levels, implying that different long-term (safe) and short-term (speculative) investors need to employ different trading strategies to hedge against or to speculate on the market's response to the pandemic. From a diversification perspective, since COVID-19 statistics tend have similar effects across all stock indices in G20 countries, it is inadvisable for market participants to diversify their portfolios amongst these markets and investors should rather seek to diversify their portfolios in other markets which are less influenced by the pandemic. In this regard, the main shortcoming of our study is that it does not identify other stock or financial markets which are less affected by COVID-19 and this can serve as an avenue for future research.

## 6 Conclusion

Our study re-examines the impact of the COVID-19 health statistics (cases, cures and deaths) on stock market returns in G20 countries using more recent data (February 2, 2020–August 28, 2021) and more specialized methodologies to simultaneously capture time and frequency variation (DCC-GARCH and wavelet coherence). Firstly, we used the DCC-GARCH framework to model the time-varying co-movement in volatility between COVD-19 statistics and equity returns. Secondly, we use wavelet coherence tools to model the time-frequency co-movement between the variables.

On one hand, the DCC models provide evidence for time-varying co-variability between the series, more specifically for recoveries and deaths, whilst those for cases are short-lived until mid-April. Moreover, the co-movements between the series frequently switches between being negative and positive. On the other hand, the wavelet coherence provides evidence on the dominance of low-frequency co-movements across the entire time window although higher frequency components are observed around periods of peaking COVID statistics and announcements of the different COVID variants. More interesting, the phase dynamics indicate that low frequency components are primarily anti-phase (negative) whereas higher frequency components are in-phase (positive).

Our study has important implications towards theory, policy practice, market behaviour and academics. For starters, our results provide support of the pandemic characterizing a Black Swan event particularly considering that co-movement between the effect of COVID-19 statistics on stock markets is frequently changing between negative and positive across both time and frequency domains. Moreover, our findings also provide support in favour of the semi-form AMH in which find that information efficiency in stock markets has been varying throughout the pandemic. For market participants, our study demonstrates that equity markets are vulnerable towards speculative behaviour during periods of panic particular during announcements of different variants of the disease where higher frequency components are most dominant. For policymakers, our study shows that health outcomes are least important for equity market outcomes and the recent urgency of governments worldwide in prioritizing the vaccination of large populations could play a role in determining market stability and efficiency. For academics, our study highlights the importance of using more updated time series and methodologies to draw new information on the subject and encourages future studies to keep monitoring the evolving nature of the pandemic on stock markets as more data becomes available.

## Author contribution statement

Andrew Phiri; Izunna Anyikwa: Conceived and designed the experiments; Performed the experiments; Analyzed and interpreted the data; Contributed reagents, materials, analysis tools or data; Wrote the paper. Clement Moyo: Conceived and designed the experiments; Contributed reagents, materials, analysis tools or data; Wrote the paper.

## Funding statement

This research did not receive any specific grant from funding agencies in the public, commercial, or not-for-profit sectors.

## Data availability statement

The authors do not have permission to share data.

## Declaration of competing interest

The authors declare no conflict of interest.

## Figures and Tables

**Table 1:** Descriptive statistics

Variables	Mean (%)	Std. Dev.	Skewness	Kurtosis	Jarque-Bera	ADF-level	PP-level	ARCH(10)	LB(10)	LB-sq(10)
Cases	2.288	5.263	6.394	55.698	68491.0***	−4.072***	−8.755***	32.656***	146.82***	264.830***
Recovery	2.723	5.612	4.790	32.260	22079.0***	−7.685***	−11.805***	192.450***	327.300***	419.960***
Death	2.223	5.131	5.100	35.880	27604.8***	−4.487***	−11.060***	92.391***	199.600***	367.030***
Argentina	0.133	2.721	−0.518	7.415	479.9***	−0.770	−0.778	152.575***	17.165*	340.180***
Australia	−0.050	1.548	−1.063	10.697	1488.0***	−0.994	−1.212	251.267***	25.833***	821.300***
Brazil	−0.117	2.415	−0.116	18.242	5422.1***	−1.228	−1.438	300.749***	19.860**	808.020***
Canada	0.045	1.743	0.019	29.115	15913.5***	−1.740	−0.924	191.126***	22.154**	511.100***
China	−0.030	1.235	−0.803	7.225	476.6***	−1.406	−1.416	70.3487***	16.815*	97.333***
France	0.013	1.641	−1.036	13.882	2863.4***	−0.907	−1.119	109.037	13.925	262.090***
Germany	0.013	1.635	−1.150	12.482	2221.3***	−0.997	−1.182	78.099***	13.802	145.500***
India	0.106	1.721	−1.239	15.104	3561.997	−0.357	−0.359	111.862***	52.171***	249.290***
Indonesia	0.027	27.288	0.530	278.840	1775408***	−7.992***	−23.362***	51.876***	83.769***	59.823***
Italy	0.011	1.772	−2.500	28.524	15785.2***	−1.209	−1.435	119.156***	18.050*	163.420***
Japan	−0.050	1.498	−0.155	6.797	338.6***	−0.841	−0.913	171.157***	28.549***	295.770***
Korea	0.087	1.559	0.073	8.580	727.1***	−0.384	−0.481	263.285***	17.778*	574.980***
Mexico	−0.035	1.397	−0.258	6.503	292.5***	−0.575	−0.552	141.357***	10.974	326.990***
Russia	−0.023	1.348	−0.958	11.577	1802.2***	−0.538	−0.712	131.519***	38.908***	318.610***
SA	0.049	1.684	−0.281	10.716	1396.7***	−1.046	−1.112	225.732***	27.077***	812.330***
Saudi	0.067	1.145	−2.011	20.177	7261.7***	−0.263	0.080	146.639***	42.954***	259.320***
Spain	−0.062	1.711	−1.246	16.423	4349.3***	−1.847	−1.977	90.840***	24.972***	164.030***
Turkey	−0.006	1.465	−1.500	11.093	1738.2***	−0.834	−0.954	33.794***	17.574*	45.162***
UK	−0.044	1.503	−1.022	12.989	2425.7***	−2.171	−2.202	97.277***	13.235	168.050***
US	0.098	1.729	−0.638	15.085	3445.8***	−0.989	−0.534	285.851***	43.462***	1036.00***

Note: ***. ** and * denote significance at 1%, 5% and 10% respectively. ADF and PP indicate the Augmented Dickey-Fuller and Phillip-Perron unit root tests results in levels. The ARCH test is the autoregressive conditional heteroscedasticity test. The LB and LB-squared are the Ljung-Box residual tests for serial correlation.

**Table 2:** Unconditional correlation

Country	Cases	Recovery	Death
Argentina	0.065	0.056	0.083
Australia	0.096	0.080	0.101
Brazil	0.094	0.090	0.097
Canada	0.094	0.081	0.104
China	0.182	0.193	0.203
France	0.118	0.099	0.125
Germany	0.109	0.086	0.120
India	0.087	0.077	0.103
Indonesia	0.003	0.003	0.004
Italy	0.106	0.089	0.112
Japan	0.125	0.100	0.135
Korea	0.115	0.101	0.129
Mexico	0.133	0.128	0.144
Russia	0.159	0.139	0.167
South Africa	0.076	0.057	0.086
Saudi Arabia	0.130	0.114	0.143
Spain	0.105	0.086	0.107
Turkey	0.095	0.084	0.111
UK	0.119	0.101	0.130
US	0.093	0.085	0.103

**Table 3:** DCC-GARCH (1, 1) test result

Country	μ	φ	ω	α	β	a + b	ARCH(10)	LQ(10)	LQ-sq(10)
Argentina	0.188	0.299***	0.427*	0.147***	0.783***	0.962	9.856	10.306	10.946
Australia	0.051	0.115**	0.084**	0.248***	0.698***	0.954	10.776	11.471	10.094
Brazil	0.129**	0.111**	0.829***	0.706***	0.147**	0.962	6.576	13.113	6.414
Canada	0.102***	0.129**	0.110***	0.569***	0.409***	0.956	3.385	22.411***	3.431
China	0.053	0.355***	0.075**	0.153***	0.789***	0.949	10.064	22.412***	10.21
France	0.079	0.169***	0.050	0.134***	0.846***	0.954	2.903	15.579*	2.992
Germany	0.100*	0.136***	0.067*	0.136***	0.837***	0.952	2.430	14.508	2.510
India	0.130*	0.229***	0.045*	0.126***	0.852***	0.955	4.775	12.735	4.764
Indonesia	0.123***	−0.896***	0.403	15.392	0.013	0.928	2.084	8.133	2.168
Italy	0.096*	0.129**	0.077*	0.163***	0.813***	0.954	7.444	12.994	7.925
Japan	0.021	0.252***	0.513***	0.394***	0.324**	0.954	6.739	21.828***	6.594
Korea	0.131**	0.262***	0.106***	0.267***	0.680***	0.951	13.177	16.008*	12.593
Mexico	0.083	0.182***	0.391***	0.378***	0.386***	0.956	8.000	15.662*	7.675
Russia	0.046	0.230***	0.028	0.080*	0.897***	0.956	12.738	16.109*	12.467
South Africa	0.080	0.265***	0.124*	0.208***	0.729***	0.953	8.855	2.719	8.671
Saudi Arabia	0.126***	0.273***	0.028*	0.121*	0.856***	0.953	2.151	6.746	2.302
Spain	0.007	0.170***	0.093*	0.150***	0.819***	0.956	4.364	11.265	4.737
Turkey	0.176***	0.156***	0.266	0.186*	0.698***	0.953	1.673	19.730**	1.662
UK	0.001	0.200***	0.044	0.107***	0.867***	0.951	11.265	14.362	11.957
US	0.152***	0.122**	0.074**	0.317***	0.673***	0.951	4.394	20.175**	4.400

Note: ***, ** and * denote statistically significant at 1%, 5% and 10% levels, respectively. a + b is the sum of the DCC parameters. Stability condition requires that a + b < 1 be satisfied. The ARCH test is the autoregressive conditional heteroscedasticity test. The LB and LB-squared are the Ljung-Box residual tests for serial correlation.

**Table 4:** Average DCC

Country	Cases	Recovery	Death
Argentina	−0.008	0.060	0.000
Australia	−0.012	0.027	0.039
Brazil	−0.025	0.003	0.036
Canada	−0.013	0.021	0.045
China	−0.033	0.025	−0.055
France	−0.001	0.038	0.015
Germany	0.002	0.029	0.036
India	−0.009	0.011	0.053
Indonesia	−0.002	0.008	0.033
Italy	0.003	0.026	0.012
Japan	−0.007	0.020	−0.003
Korea	−0.027	−0.013	0.024
Mexico	−0.025	−0.006	−0.022
Russia	−0.006	0.014	0.078
South Africa	−0.002	−0.021	0.040
Saudi Arabia	−0.014	0.053	−0.011
Spain	0.000	0.051	0.019
Turkey	−0.006	−0.014	0.047
UK	−0.001	0.066	0.035
US	−0.007	−0.008	−0.005
